# Characteristics of the Early Immune Response Following Transplantation of Mouse ES Cell Derived Insulin-Producing Cell Clusters

**DOI:** 10.1371/journal.pone.0010965

**Published:** 2010-06-04

**Authors:** Ashleigh S. Boyd, Kathryn J. Wood

**Affiliations:** 1 Transplantation Research Immunology Group, Nuffield Department of Surgery, John Radcliffe Hospital, University of Oxford, Oxford, United Kingdom; 2 NIH Center of Biomedical Research Excellence (COBRE) in Tissue Repair and Stem Cell Biology, Roger Williams Hospital, Boston University School of Medicine, Providence, Rhode Island, United States of America; Albert Einstein Institute for Research and Education, Brazil

## Abstract

**Background:**

The fully differentiated progeny of ES cells (ESC) may eventually be used for cell replacement therapy (CRT). However, elements of the innate immune system may contribute to damage or destruction of these tissues when transplanted.

**Methodology/Principal Findings:**

Herein, we assessed the hitherto ill-defined contribution of the early innate immune response in CRT after transplantation of either ESC derived insulin producing cell clusters (IPCCs) or adult pancreatic islets. Ingress of neutrophil or macrophage cells was noted immediately at the site of IPCC transplantation, but this infiltration was attenuated by day three. Gene profiling identified specific inflammatory cytokines and chemokines that were either absent or sharply reduced by three days after IPCC transplantation. Thus, IPCC transplantation provoked less of an early immune response than pancreatic islet transplantation.

**Conclusions/Significance:**

Our study offers insights into the characteristics of the immune response of an ESC derived tissue in the incipient stages following transplantation and suggests potential strategies to inhibit cell damage to ensure their long-term perpetuation and functionality in CRT.

## Introduction

The capacity of embryonic stem cells (ESC) to form multiple tissue types has fuelled hope that they may eventually be used to provide an alternative or supplementary supply of tissue for cell replacement therapy (CRT) in diseases that lead to organ degeneration or failure, such as Type 1 *diabetes mellitus* [1]. However, the host immune response invoked following transplantation of ESC derived tissue presents a potential impediment to their therapeutic use [2], [3].

Use of an ESC derived tissue in CRT may be limited by a number of generic events that impinge on the functionality of transplanted tissue. Firstly, any episode inducing tissue damage, such as the process of transplantation, will elicit an early inflammatory response, even in the syngeneic setting [4], [5]. While this complex, multi-factorial response to injury has evolved to protect the host against pathogens, rejuvenate damaged tissue and restore homeostasis, acute inflammation could be damaging to transplanted tissue and may be a crucial factor in determining optimum graft functionality; this issue has been hypothesized to be of importance in graft function in islet transplantation [6], [7]. Secondly, an early inflammatory response may provide the foundation for activation of an antigen specific, adaptive immune response in an allograft setting [8]. In this respect, mounting evidence suggests the adaptive immune response may be invoked as a direct corollary of an inflammatory response [9]. Thus, in addition to the potential damage to transplanted tissue caused by inflammation, the early immune events after transplantation may also impact rejection of transplanted tissue in the longer term.

Studies hitherto have almost exclusively focused on the adaptive immune response toward ESC or ESC derived allografts [10], [11], [12] and the early immune response towards transplanted ESC derived tissue has largely been neglected. In addition, an assessment of the immunogenicity of terminally differentiated ESC products has been lacking; this is a critical issue as undifferentiated ESC and terminally differentiated ESC progeny can exhibit differing immunogenicity [2], [13]. By comparing the immune response following either implantation of ESC derived insulin producing cell clusters (IPCC) or adult pancreatic islets of Langerhans, we have therefore assessed the early immune response to fully differentiated ESC tissue during the first three days following transplantation of either syngeneic or allogeneic tissue.

## Materials and Methods

### ESC culture and differentiation to insulin producing cell clusters (IPCCs)

The ESC line ESF 122 was maintained as described previously[1]. Briefly, ESC were plated onto mitotically inactivated primary embryonic fibroblasts (3000 rad) in ESC medium composed of knock-out (KO-) DMEM (Invitrogen, Paisley, Scotland), 15% FCS, 1% 100 µM L-glutamine, 1% non-essential amino acids (non-eAAs) (all Invitrogen), 1% 100 µM penicillin-streptomycin, 100 µM β-ME and 100 µl/10ml medium 10 µg/ml LIF (Chemicon International, Temecula, California, USA). Directed differentiation of ESC was achieved using a modified form of the Blyszczuk protocol as described previously [1]. See Supplemental Figure S1 for further details.

### Animals

7-12 week old female syngeneic CBA or allogeneic C57 BL/10 mice were obtained from and housed within the Biomedical Services Unit (BMSU) of the John Radcliffe Hospital (Oxford, UK).

### Ethics Statement

The ESC line ESF 122 was generated as described previously [1]. Mice were maintained *ad libitum* on sterilised food and water in accordance with the animal care and use guidelines approved by the Home Office (London, UK).

### Isolation of adult pancreatic islets

Adult pancreatic islets were isolated as described previously [1]. Briefly, islets were isolated by collagenase digestion of the pancreas, followed by centrifugation through a discontinuous Ficoll gradient.

### Transplantation of IPCCs or pancreatic islets

Transplantation of IPCCs or pancreatic islets was performed as described previously [1]. Briefly, 300 IPCCs or pancreatic islets were transplanted under the sub-capsular renal space of an anesthetized mouse. ‘Sham’ transplantation was performed as a control for the non-specific inflammation induced by the surgical procedure itself; the recipients received no cells, but underwent the surgical procedure: the abdomen of each anesthetized mouse was opened, the kidney exposed and an incision made in the kidney capsule. Post-transplant, vicryl sutures were used to close the peritoneum and the skin of all transplanted recipients.

### Immunofluorescence

Islet and IPCC subcapsular kidney grafts were harvested, embedded in OCT Tissue-Tek (Miles Diagnostics, Elkhart, IN, USA) compound. 6 µm sections were air dried and then fixed in acetone. Non-specific binding was blocked with PBS/10% FCS or PBS/4% mouse, goat or rabbit serum, depending on the primary antibody used, prior to primary antibody staining with Gr-1, Mac-1 or C3. Following secondary antibody staining, the sections were mounted with Vectashield mounting medium with 4′, 6-diamino-2-phenylindole (DAPI) (Vector Labs, UK) and visualized with a Zeiss Fluorescence microscope using Openlab 4.0.1 software.

### Quantitative real-time PCR

RNA and cDNA preparation was performed as described previously [1]. Briefly, total cellular RNA was isolated from undifferentiated ES cells, end-stage IPCCs, pancreas, islets and spleen using the Stratagene Absolutely RNA™ kit. 2ng of RNA, was reverse transcribed into cDNA with Moloney Murine Leukaemia Virus (MMLV)-reverse transcriptase (MMLV-RT, Invitrogen) according to the manufacturers instructions. 2.5ng of template was used and for each sample PCR was performed in duplicate or triplicate. PCR primer sets used in this study have been previously validated and sequences are provided in Supplemental Table S1 and S2 [4], [14]. Quantitation was performed by standard methods described previously [15].

### Statistical analysis

Cellular infiltration and PCR data were subjected to statistical analyses. The paired, 2-tailed Student *t* test was used to compare two biologically paired samples transplanted under the same conditions into age and sex matched recipients

## Results

### Experimental model

We have previously compared multiple methods for the differentiation of ESC to IPCCs and confirmed the fully differentiated status of IPCCs [1], [13]. By using a modified protocol for ESC differentiation to IPCCs derived from Blyszczuk et al [1], [16] (**Supplemental Figure S1**), we now assess the early immune events that follow transplantation of IPCCs derived from ESF 122 ESC (H2^k^) or adult pancreatic islets isolated from CBA mice (H2^k^) [1], [2], [13] (**Supplemental Figure S2**). The immune response to transplanted tissue was evaluated in a non-diabetic mouse background to avoid the confounding contribution of the autoimmunity observed in diabetic mice or mice with chemically induced diabetes in vivo. Three hundred IPCCs or adult pancreatic islets were transplanted under the kidney capsule of syngeneic (CBA) or allogeneic (C57 BL/10, H2^b^) recipient mice and the graft was excised 1 or 3 days later. Half of the kidney containing the transplanted cells was reserved for immunofluorescence staining and histological examination, while the other half was analysed by quantitative real-time PCR (Q-PCR) analysis of gene expression. Importantly, ‘sham’ transplantation was performed as a control for the non-specific inflammation caused by the surgical procedure used for tissue implantation. Assessing immune cell infiltration or relative gene expression against ‘sham’ transplant controls therefore affords a more accurate assessment of the immune response induced specifically by transplantation of syngeneic versus allogeneic IPCC tissue.

### IPCC allografts are less prone to infiltration by innate immune cells early after transplantation

**Figure 1:**
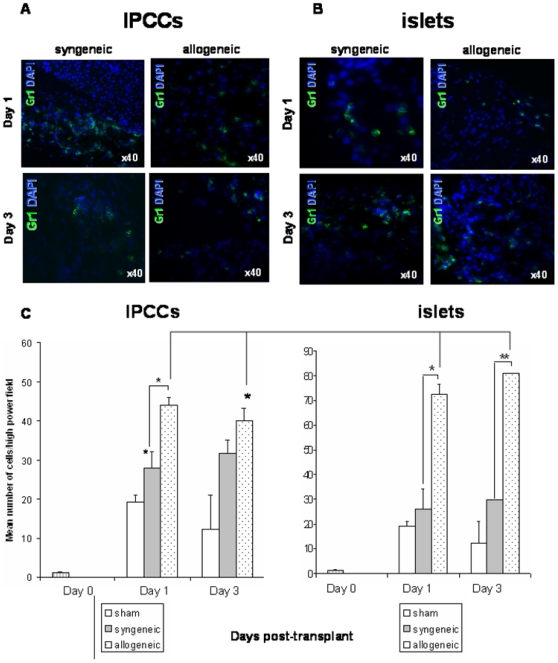
IPCC allografts are less prone to neutrophil infiltration early after transplantation. (**A**) 300 IPCCs generated from CBA-derived ES cells or (**B**) 300 adult pancreatic islets were transplanted under the kidney capsule of syngeneic (CBA, H2^k^) or allogeneic (C57 BL/10, H2^b^) recipient mice and the graft harvested either on day 1 or 3 days post-transplant. Sections were stained for the granulocyte marker Gr1/Ly6G (FITC, green) and counterstained with the DAPI nuclear-dye (blue) (n = 4 experiments). Sham transplantations were also performed (data not shown). The spleen served as positive staining control (Supplemental Figure 3). Photographs were taken using a 40x objective and a 10x eyepiece lens, giving a total original magnification of ×400. (**C**) The number of Gr1^+^ cells infiltrating IPCC grafts or islet grafts was quantified by counting the number of green cells in 5 high power fields per sample (n = 4−6 independent samples/time point from independent differentiations). A naïve kidney was harvested from an un-manipulated mouse as a negative control for Gr1^+^ cells within the kidney at day 0 (striped bar). Data shown are the mean ± SEM and statistical significance was assessed by unpaired Student's t test and denoted by an asterisk in the figure.

We firstly examined innate leukocyte infiltration to the IPCCs or islet in the initial stages after transplantation. In comparison to ‘sham’ transplant controls, neither syngeneic IPCCs or syngeneic adult islets induced infiltration of Gr1^+^ cells within the first 24 hours after implantation (**Figure 1A, 1B and 1C** **; for positive controls see Supplemental Figure S3)**. However, at the same time point, the number of Gr1^+^ cells infiltrating was noticeably higher in allogeneic IPCC grafts compared with both syngeneic transplants and sham control transplants (**Figure 1A and 1C**). Compared to either syngeneic islet grafts or the ‘sham’ transplantation at day 1 and 3, a significant Gr1^+^ infiltrate was noted at the site of implantation of allogeneic islet grafts (**Figure 1B and 1C**). These data show that the early infiltration of Gr-1^+^ cells occurs exclusively in the setting of allogeneic IPCC or islet transplantation. Of interest, however, IPCC allografts were infiltrated by Gr-1^+^ cells to a lesser extent than islet allografts at three days after transplantation, showing that ES cell derived IPCCs may be less to susceptible to immune attack by neutrophils than adult islet tissue (**Figure 1C**).

**Figure 2:**
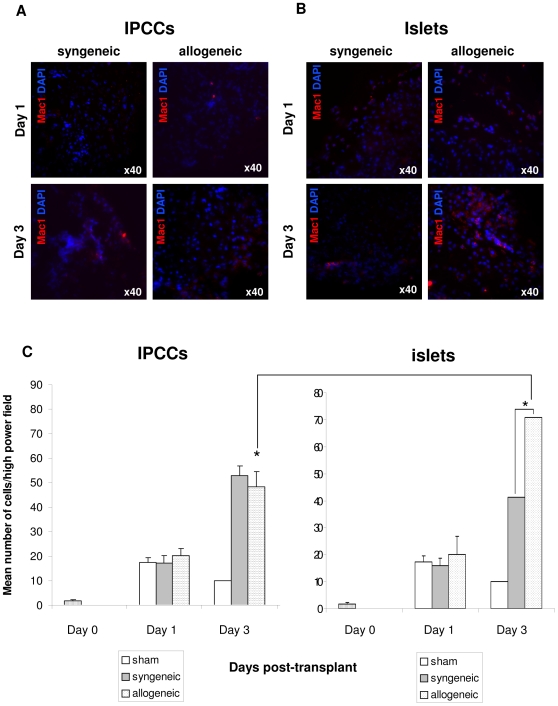
Attenuated influx of macrophages to IPCC allografts early after transplantation. (**A**) 300 IPCCs generated from CBA-derived ES cells or (**B**) 300 adult pancreatic islets were transplanted under the kidney capsule of syngeneic (CBA, H2^k^) or allogeneic (C57 BL/10, H2^b^) recipient mice and the graft harvested either on day 1 or 3 days post-transplant. Sections were stained for the granulocyte marker Mac-1/CD11b (red) and counterstained with the DAPI nuclear-dye (blue) (n = 4 experiments). (data not shown). Sham transplantations were also performed (data not shown). The spleen served as positive staining control (Supplemental Figure 3). Photographs were taken using a 40x objective and a 10x eyepiece lens, giving a total original magnification of ×400. (**C**) The number of Mac-1^+^ cells infiltrating IPCC grafts or adult islet grafts was quantified by counting the number of green cells in 5 high power fields per sample (n = 4−6 independent samples/time point from multiple independent differentiations). A naïve un-transplanted kidney was harvested from an un-manipulated mouse as a control to represent the baseline level of Mac-1^+^ cells within the kidney at day 0 (striped bar). Data shown are the mean ± SEM and statistical significance was assessed by unpaired Student's t test and denoted by an asterisk in the figure.

Monocyte/macrophage infiltration, as judged by CD11b staining, was comparable between ‘sham’ transplanted and IPCC syngeneic or allogeneic graft sites at day 1 (**Figure 2A and 2C**). On day 3, however, both the syngeneic and allogeneic grafts were infiltrated by Mac-1^+^ cells above levels observed in ‘sham’ controls and the abundance of infiltrating cells was similar in each case (**Figure 2A and 2C**). Likewise, a similar degree of infiltration by CD11b^+^ cells was noted in syngeneic and allogeneic islet grafts and ‘sham’ transplanted animals at day 1 (**Figure 2B and 2C**). However, by day 3 islet grafts provoked an increased degree (2-fold) of infiltration by CDl1b^+^ cells than the ‘sham’ control (**Figure 2B and 2C**). As observed for infiltration of Gr1^+^ cells, IPCC allografts were infiltrated by fewer CD11b^+^ cells than islet allografts at three days after transplantation (**compare** **Figures 1C** **and** **2C**).

Jointly these data indicate that in the first 3 days following implantation innate immune cells can infiltrate the graft site as a specific response to the presence of the transplanted IPCCs and across allogeneic barriers. Intriguingly, however, these data demonstrate that at this early time after transplantation, IPCC allografts are less prone to infiltration by innate immune cells than islet allografts.

### Attenuated infiltration of innate immune cells across IPCC allogeneic barriers was independent of complement deposition

**Figure 3:**
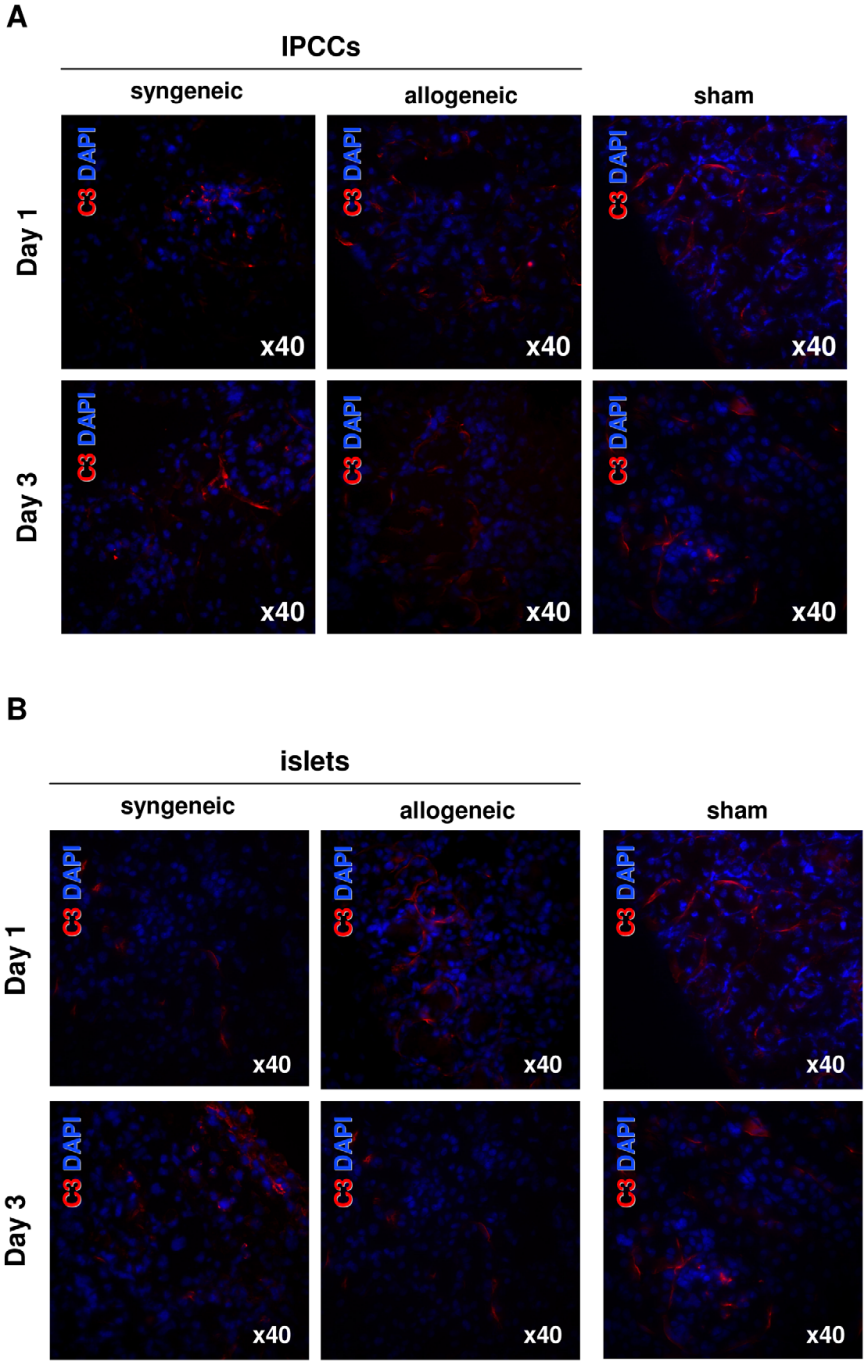
Attenuated infiltration of innate immune cells across IPCC allogeneic barriers was independent of complement deposition. (**A**) 300 IPCCs generated from CBA-derived ES cells or (**B**) 300 adult pancreatic islets were transplanted under the kidney capsule of syngeneic (CBA, H2^k^) or allogeneic (C57 BL/10, H2^b^) recipient mice and the graft harvested either on day 1 and 3 days post-transplant. Sham transplantations were also performed. Sections were stained for the complement molecule C3 (red) and counterstained with the DAPI nuclear-dye (blue) (n = 3 independent experiments at each time point). Photographs were taken using a 40x objective and a 10× eyepiece lens, giving a total original magnification of ×400.

The previous data indicates that activation of the innate immune system occurs after transplantation of IPCCs across allogeneic barriers, albeit to a lesser extent than in islets. Since the complement system is a critical bridge between the innate and adaptive immune responses [17], we next chose to examine whether the complement molecule C3, a molecule central to the activation of all 3 complement pathway cascades, was detectable in IPCC or islet transplanted recipients. **Figure 3A** shows sections of IPCC engrafted kidney staining strongly for C3 in both syngeneic and allogeneic groups; C3 was observed in sections excised 1 day after transplant and a robust C3 signal was retained on day 3. The transplanted islet samples (**Figure 3B**) demonstrated a similar strength signal and expression pattern of C3 in comparison to IPCC on day 1 until day 3 post-transplant sections. Of particular note, examination of the structure of the kidney versus grafted tissue demonstrated that C3 was bound indiscriminately throughout the kidney, with no differences observed between sample types. Interestingly, the ‘sham’ transplantation showed similar staining for C3 compared to either islets or IPCCs in a syngeneic or allogeneic background (**Figure 3A and B**). Taken together, these data show that complement activation resulting in the production of complement fragments that bind covalently to IPCCs or islets occurs regardless of the presence of the transplanted tissue itself. These data also imply that attenuated infiltration of innate immune cells across IPCC allogeneic barriers occurs independently of complement activation.

### Marked reduction in IL-6 at the early stages following IPCC transplantation

**Figure 4:**
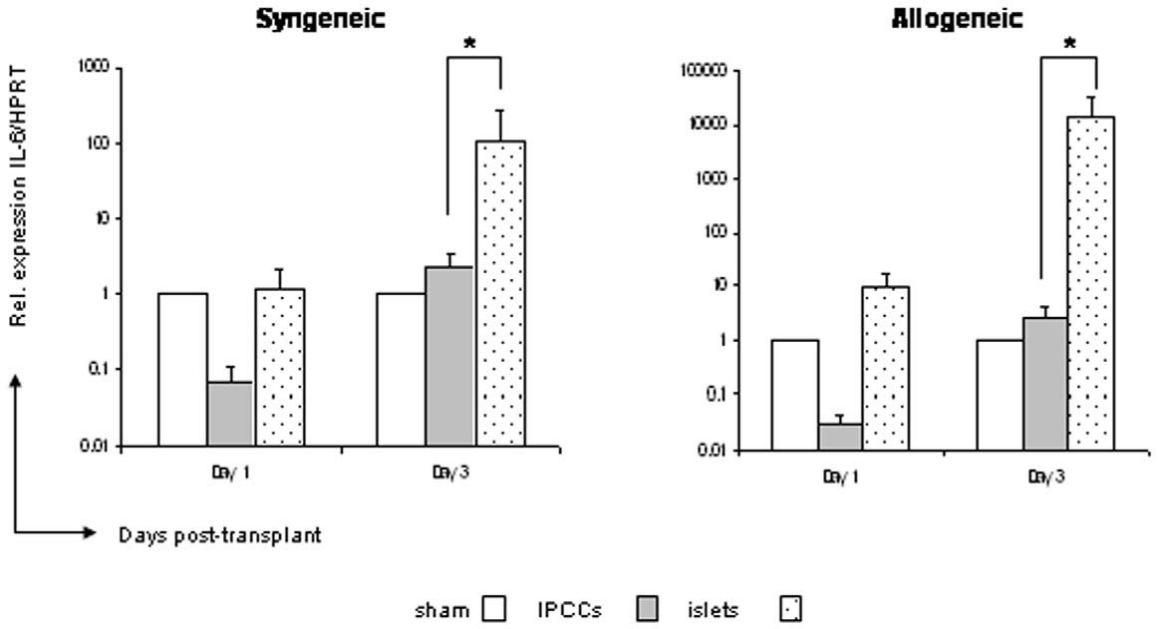
Marked reduction in IL-6 at the early stages following IPCC transplantation. 300 IPCCs generated from CBA-derived ES cells or 300 adult pancreatic islets isolated from CBA mice were transplanted under the kidney capsule of B10 (H2^b^, allogeneic) and CBA (H2^k^, syngeneic) recipients. Intra-graft mRNA expression for the cytokine IL-6, was analysed at days 1 and 3 (n = 6−9 mice per time point from multiple differentiations) after transplantation by Q-PCR. Samples were standardised against HPRT. Data shown are the mean ± SEM and statistical significance was assessed by unpaired Student's t test and denoted by an asterisk in the figure.

As early inflammation at the site of transplantation can lead to the generation and release of chemical mediators, such as cytokines [18], we assessed the production of inflammatory cytokines IL-6 and TNF-α after transplantation of IPCCs or islets into syngeneic or allogeneic recipients by Q-PCR. When IL-6 mRNA expression was analysed at 3 days after transplantation, a 100 and 10000 fold increase in IL-6 mRNA expression was noted in syngeneic and allogeneic islet samples respectively. Importantly, IL-6 mRNA was not up-regulated by day 3 in either IPCC syngeneic grafts or allografts when compared to ‘sham’ transplantation (**Figure 4**). In contrast, TNF-α expression was not observed in the early immune response to either IPCC or islet transplantation (**data not shown**). Ergo, in contrast to islet transplantation, IL-6 mRNA was not produced specifically as an early inflammatory response to IPCC transplantation.

### Attenuated inflammatory chemokine expression immediately following IPCC transplantation

**Figure 5:**
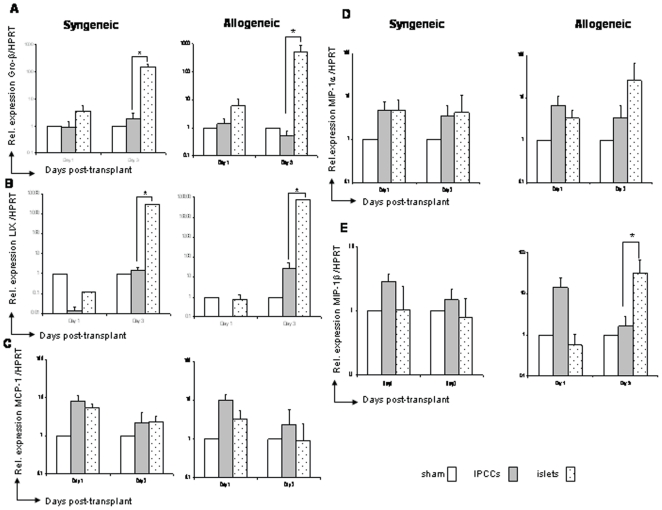
The expression of specific inflammatory chemokines was either absent or sharply reduced at the early stages after syngeneic or allogeneic IPCC transplantation. 300 IPCCs generated from CBA-derived ES cells or 300 adult pancreatic islets isolated from CBA mice were transplanted under the kidney capsule of B10 (H2^b^, allogeneic) and CBA (H2^k^, syngeneic) recipients. Intra-graft mRNA expression for inflammatory chemokines (**A**) Gro-β/CXCL2, (**B**) LIX/CXCL5, (**C**) MCP-1/CCL2, (**D**) MIP-1α/CCL3 and (**E**) MIP-1β/CCL4 were analysed at days 1 and 3 (n = 6−9 mice per time point from multiple differentiations) after transplantation by Q-PCR. Samples were standardised against HPRT. Data shown are the mean ± SEM and statistical significance was assessed by unpaired Student's t test and denoted by an asterisk in the figure.

Inflamed transplanted tissues can themselves produce an ‘early’ wave of chemokines, including Gro-β/CXCL2, Lix/CXCL5, MCP-1/CCL2, MIP-1α/CCL3 and MIP-1β/CCL4, which can mediate the migration of immune cells to the site of tissue damage or injury [9], [19]. By Q-PCR, we next examined the expression of these chemokines up to three days following IPCC or islet transplantation. After IPCC transplantation, MCP-1/CCL2, MIP-1α/CCL3 and MIP-1β/CCL4 mRNA were induced above the level detected in the ‘sham’ transplantation controls at day 1, indicating that IPCCs specifically induce these chemokines immediately after transplantation (**Figure 5C-5E**). Excepting the lack of induction of MIP-1β/CCL4 in islet syngeneic and allogeneic grafts, these results were broadly comparable to those obtained following transplantation of islets into syngeneic or allogeneic mice at day 1 (**Figure 5E**). At day 3, weak or no induction of Gro-β/CXCL2, MCP-1/CCL2, MIP-1α/CCL3 and MIP-1β/CCL4 mRNA was noted in syngeneic or allogeneic IPCC grafts compared to the ‘sham’ transplantation (**Figure 5A, 5C-E**). Only expression of MCP-1/CCL2 mRNA was similarly low in syngeneic and allogeneic transplanted islets at day 3 (**Figure 5C**). Moreover, the production of specific ‘inducible inflammatory’ chemokines at the site of implantation of IPCCs was reduced in comparison to that following islet transplantation (**Figure 5A, 5B and 5E**). In particular, Gro-β/CXCL2 and Lix/CXCL5 were massively down-regulated in IPCCs compared to islet grafts in both the syngeneic and allogeneic setting (150-fold increase for the syngeneic islet group and 504-fold increase for the allogeneic islet group for Gro-β/CXCL2 and 1000 fold increase in the syngeneic islet group and 100000 fold in the allogeneic islet group for Lix/CXCL5) (**Figure 5A and 5B**). Of the other chemokines assessed, MIP-1β/CCL4 mRNA was significantly reduced in IPCCs compared with islets transplanted into allogeneic recipients at day 3 (**Figure 5E**). A lower, non-statistically significant attenuation of MIP-1α/CCL3 mRNA was also observed in the IPCCs compared with islets transplanted across the same allogeneic barrier at day 3 (**Figure 5D**). Parenthetically, the relative reduction of inflammatory cytokines and chemokines after IPCC transplantation occurs independently of heme-oxygenase-1, a stress response protein that alleviates inflammation and oxidative stress [20] (**data not shown**). These data collectively demonstrate that in comparison to islet grafts, IPCCs induced substantially less inflammatory chemokine expression after three days of transplantation in the syngeneic and/or allogeneic setting.

### Expression of IP-10/CXCL10, a chemokine associated with acute graft rejection, was reduced after IPCC transplantation

**Figure 6:**
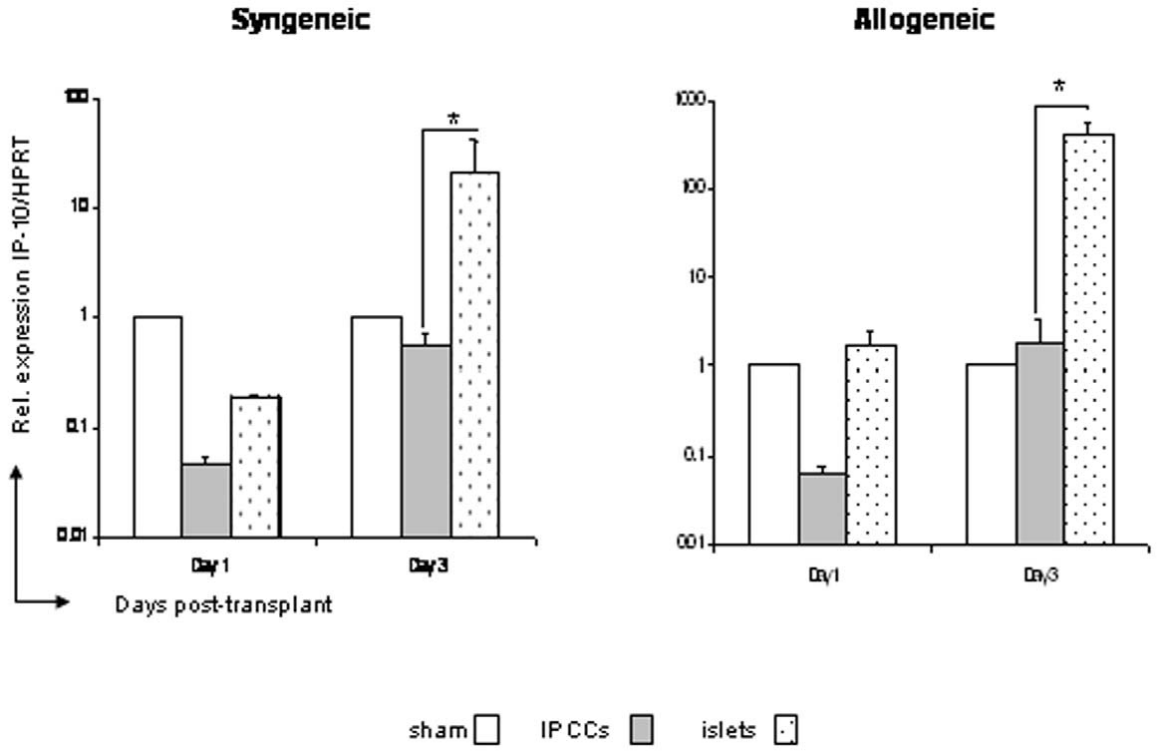
IP-10/CXCL10, a chemokine associated with acute graft rejection, was reduced immediately following IPCC transplantation. 300 IPCCs generated from CBA-derived ES cells or 300 adult pancreatic islets isolated from CBA mice were transplanted under the kidney capsule of B10 (H2^b^, allogeneic) and CBA (H2^k^, syngeneic) recipients. Intra-graft mRNA expression of a chemokine associated with rejection IP-10/CXCL10 was analysed at days 1 and 3 (n = 6−9 mice per time point from multiple differentiations) after transplantation by Q-PCR. Samples were standardised against HPRT. Data shown are the mean ± SEM and statistical significance was assessed by unpaired Student's t test and denoted by an asterisk in the figure.

The chemokines IP-10/CXCL10 and Mig/CXCL9 are often associated with acute rejection in transplantation [21] and we assessed their contribution to the early immune response following IPCC transplantation. Mig/CXCL9 expression was similar in IPCC or islet transplantations at day 1 and 3 in either the syngeneic or allogeneic groups and was not expressed significantly above the level observed in ‘sham’ transplantation (**data not shown**). IP-10/CXCL10 mRNA expression was similar between the ‘sham’ transplantation, IPCC and islet grafts at day 1 in the syngeneic and allogeneic groups (**Figure 6**). However, on day 3 the IPCC graft expression of IP-10/CXCL10 mRNA was very low compared with that observed in both islet syngeneic and allogeneic grafts (**Figure 6**). Incidentally, a notable increase in IP-10/CXCL10 expression was also observed between the syngeneic and the allogeneic islet transplants themselves (**Figure 6**). Thus, in comparison to islet grafts, IPCC syngeneic and allogeneic grafts demonstrated reduced expression of a single chemokine associated with graft rejection in the incipient stages following transplantation.

## Discussion

In this report, we have captured the initial events in the immune response triggered by the implantation of ESC derived IPCCs. While an early immune response was observed following IPCC transplantation, importantly, we also found that ESC derived IPCC were less immunogenic than their adult pancreatic islet counterparts, as judged by the attenuated leukocyte infiltration and generation of specific inflammatory cytokines and chemokines in the first 3 days after transplantation. That IPCCs show reduced immunostimulatory capabilities at this stage is further supported by our recent data showing the absence of up-regulation of the MHC transplantation antigens at three days following transplantation [13]. In combination, these data suggest the basis for the relative immune privilege observed in ESC derived tissue after transplantation [2], [13].

Since it is generally accepted that the innate immune system lacks antigen-specificity, one would expect to observe little variation between the events triggered by transplantation of syngeneic and allogeneic IPCCs at these very early time points. Yet an unexpected, recurring theme in this study was the rapid involvement of distinct allogeneic responses by 3 days after transplantation. Our findings are congruent with studies in other transplantation models [6], [22] and may be explained by the emerging but still under-appreciated concept of cross-talk between the innate and adaptive immune responses [23], [24]. The non-specific inflammatory response that results from the injury caused by introducing the graft into the recipient's body may be unresolved in the presence of additional immune stimuli, such as that observed in the transplantation of allogeneic ESC derived tissue. This idea is supported by our observation of sustained Gr-1^+^ and Mac-1^+^ cell infiltration at 1 week following transplantation of allogeneic, but not syngeneic IPCCs (A.S.B and K.J.W, unpublished observations). We posit that this effect may be mediated by certain effector pathways which bridge the innate and adaptive immune system [6]. Our study sets the stage for further investigations into how these two arms of the immune system conspire to impact ESC derived IPCC transplantation.

So how can we account for the ability of ESC derived IPCCs to generate less of an early immune response than adult islet grafts in the allogeneic setting? One explanation is that islets may contain passenger leukocytes that could activate the direct pathway of allorecognition [25], [26], [27]. In contrast, IPCCs are generated *in vitro* from ESC and therefore will not share the same potential as islets for co-transferal of donor-derived leukocytes. Indeed our studies examining later events involving components of the adaptive immune system after implantation of IPCCs clearly demonstrate that priming of an adaptive immune response via the presentation of allopeptides by host antigen presenting cells, the so-called indirect pathway of allorecognition, is required before a T cell response is stimulated [2]. Alternatively, islets may express more adhesion molecules such as ICAM-1 that can interact with recipient immune cells enabling them access to the graft site to intensify inflammation [28].

We found that attenuated infiltration of innate immune cells across IPCC allogeneic barriers was independent of complement deposition, as judged by the relative parity in C3 deposition between IPCCs or islets and ‘sham’ transplantation. The kidney, the site of IPCC or islet transplantation in this study, is a known producer of complement in homeostasis and injury [29], [30] and this probably accounts for the robust C3 staining observed in the sham transplantation setting. That complement deposition was not differentially enhanced by transplantation of ESC derived IPCCs or islets reflects the fact that the complement pathway was not specifically activated in response to transplantation of these tissues. However, in the longer-term, ESC allografts have been shown to be susceptible to specific activation of complement [31], which could be a direct result of the potentiation of this non-specific immune response by the adaptive immune system [9], [32]. Thus, blocking complement mediated immunogenicity could prove beneficial in ameliorating non-specific inflammatory graft damage that may be exacerbated in the presence of IPCC allogeneic tissue [33].

In the syngeneic setting, cellular infiltration and limited expression of inflammatory cytokines and chemokines was observed in IPCC grafts by day 3 (see also [13] for intra-graft IFN-γ production after syngeneic IPCC transplantation). These data broadly support our postulate that early graft damage, at the level of innate cell and inflammatory immune responses, may play a pivotal role in the later rejection of IPCCs in syngeneic, immunocompetent hosts [2]. Our data [2], [13] in the syngeneic transplant background could be pertinent when considering the use of immune matched induced pluripotent stem (iPS) cells in cell replacement therapy; iPS cells may also be subject to similar mechanisms of damage and, ultimately, rejection after transplantation even though, *a priori*, they should be histocompatible. For example, will iPS cells that have been differentiated to a specific tissue express immunogenic antigens that mark the graft for rejection*?* This is a salient issue as even minor antigen mismatches are sufficient to induce ESC graft rejection [12]. Also, how will iPS graft functionality be impaired by the infiltration of neutrophils and macrophages following transplantation? These and other associated issues warrant further evaluation.

In closing, our study suggests a potential basis for the relative immune privilege exhibited by fully differentiated ESC derived IPCCs [2], [13]. Our data also offer insights into the eminently targetable pathways that may be used to obviate the graft rejection that occurs following IPCC transplantation [2]. In this respect, it is interesting to speculate that early and sustained targeting of the innate immune system, inflammatory cytokines and/or chemokine pathways identified here may provide a specific means to achieve ESC derived IPCC graft acceptance.

## Supporting Information

Figure S1 Scheme for directed differentiation of ES cells to insulin-producing cell clusters. Starting post-embryoid body (EB) generation, hanging drop cultures (not shown) were inverted and the plate flooded with medium to suspend the EBs. 5 days after, the cells were replated onto gelatin-coated dishes for another 7 days culture in basic ES cell medium (knock-out (KO-) DMEM (Invitrogen, Paisley, Scotland), 15% FCS, 1% 100 microM L-glutamine, 1% non-essential amino acids (non-eAAs) (all Invitrogen), 1% 100 micoM penicillin-streptomycin and 100 microM beta-ME). The cell clusters were transferred onto dishes coated with poly-L-ornithine (PLO) and laminin in B2 medium, made up in DMEM: F12 (1∶1) plus N2 supplement (Sigma) and B27 supplement (Sigma). The cells were expanded in this medium for 19 days and were harvested at this point. Refer to Boyd et al., 2008 for further detailed information.
(0.01 MB PDF)

Figure S2 Experimental model used to investigate the early immune response that may arise after transplantation of either IPCCs or pancreatic islets in mice. IPCCs were generated using ESF 122 ES cells using the modified Blyszczuk protocol (See Figure S1). 300 IPCCs or 300 pancreatic islets isolated from CBA mice were transplanted under the kidney capsule of syngeneic (CBA, H2k) or allogeneic BL/10, H2b) recipient mice and the grafts excised on day 1 or 3 days post-transplant. Half the graft was taken for immunofluorescence and half to analyse intra-graft gene expression by Q-PCR.
(0.45 MB PDF)

Figure S3 The spleen is a source of Gr1+ and Mac1+ cells. In the study, spleen sections were used as a positive control for staining of Gr-1 and Mac-1 as the spleen is known to contain neutrophils and macrophages within the area called red pulp, labelled R in panel A. Staining of Gr-1 within the red pulp was evident at x100 (A) and x400 (B) original magnifications (original magnification calculated from use of a 10x eyepiece and a 40x objective lens). The area of the photograph labelled W in panel A corresponds to the T and B cell zone of the spleen, also called the white pulp. An arrow in B points to a close up of a Gr-1+ cell with a multi-lobed nucleus. Mac-1 staining can also be seen clearly at x10 (C) and x40 (D) original magnifications. These photographs are representative of n = 8 experiments.
(0.31 MB PDF)

Table S1 Primer sequences for SYBR Green Q-PCR.
(0.03 MB DOC)

Table S2 Primer sequences for TaqMan Q-PCR.
(0.03 MB DOC)
