# Supplementary material for: Characteristics of the Early Immune Response Following Transplantation of Mouse ES Cell Derived Insulin-Producing Cell Clusters
Source: PLoS One. 2010 Jun 4;5(6):e10965. doi: 10.1371/journal.pone.0010965 (PMC2881030; doi:10.1371/journal.pone.0010965)
Supplement: Figure S1 — Scheme for directed differentiation of ES cells to insulin-producing cell clusters. Starting post-embryoid body (EB) generation, hanging drop cultures (not shown) were inverted and the plate flooded with medium to suspend the EBs. 5 days after, the cells were replated onto gelatin-coated dishes for another 7 days culture in basic ES cell medium (knock-out (KO-) DMEM (Invitrogen, Paisley, Scotland), 15% FCS, 1% 100 microM L-glutamine, 1% non-essential amino acids (non-eAAs) (all Invitrogen), 1% 100 micoM penicillin-streptomycin and 100 microM beta-ME). The cell clusters were transferred onto dishes coated with poly-L-ornithine (PLO) and laminin in B2 medium, made up in DMEM: F12 (1∶1) plus N2 supplement (Sigma) and B27 supplement (Sigma). The cells were expanded in this medium for 19 days and were harvested at this point. Refer to Boyd et al., 2008 for further detailed information. (0.01 MB PDF) [file pone.0010965.s001.pdf]

Flood EB culture plate with medium

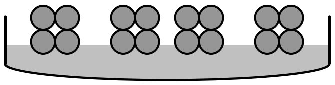

ES cell medium

3 days

Transfer EBs to gelatin-coated culture dishes

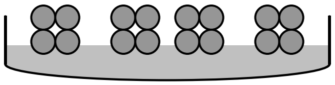

ES cell medium

7 days

Transfer clusters to PLO + laminin-coated culture dishes

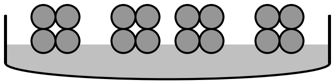

N2 medium + B27, laminin,
insulin & nicotinamide

19 days

Begin to harvest cells

Supplemental Figure 1
