# Supplementary figures and images for: Characteristics of the Early Immune Response Following Transplantation of Mouse ES Cell Derived Insulin-Producing Cell Clusters
Source: PLoS One. 2010 Jun 4;5(6):e10965. doi: 10.1371/journal.pone.0010965 (PMC2881030; doi:10.1371/journal.pone.0010965)

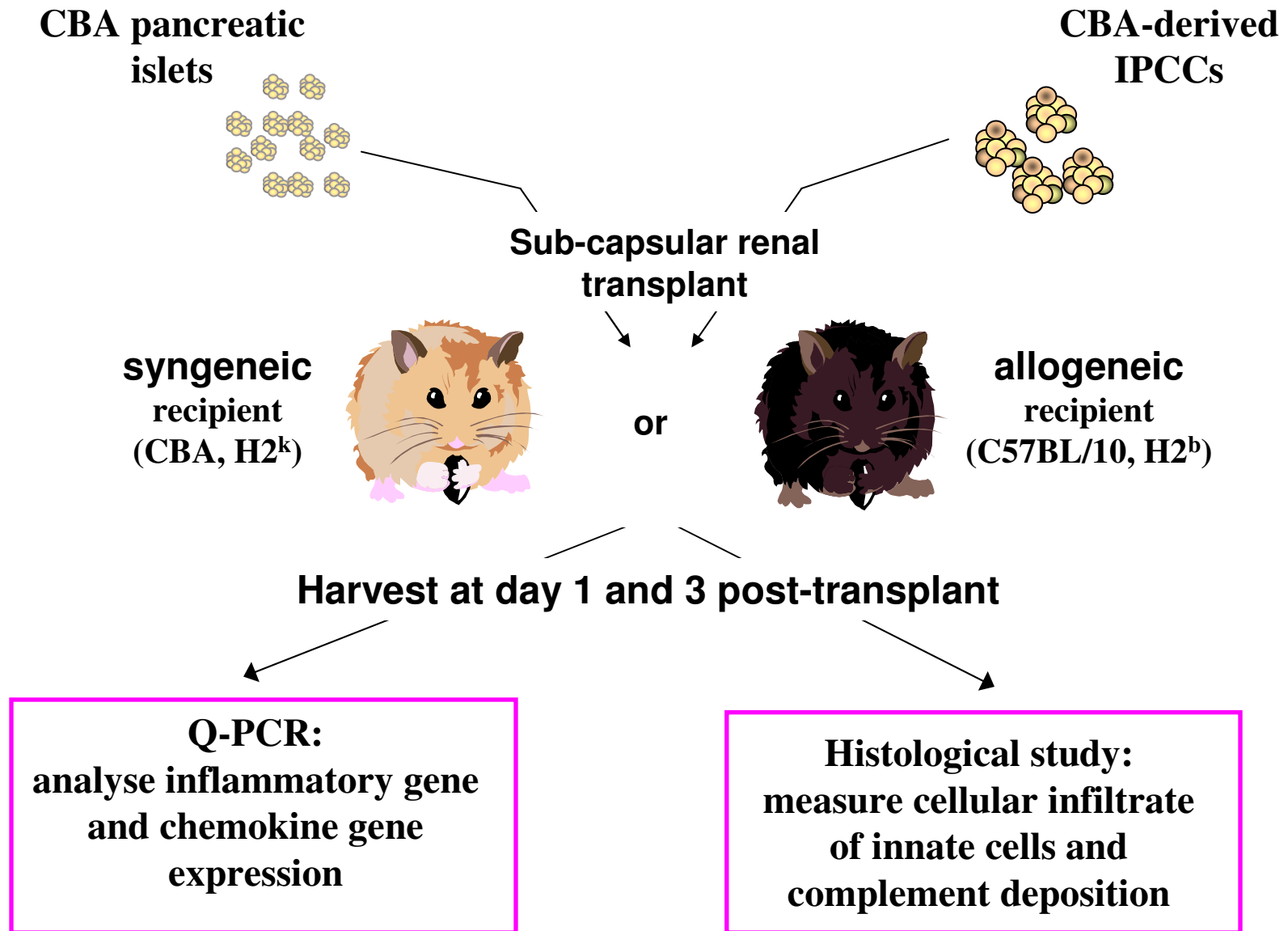

***Supplemental Figure 2***

Supplement: Figure S2 — Experimental model used to investigate the early immune response that may arise after transplantation of either IPCCs or pancreatic islets in mice. IPCCs were generated using ESF 122 ES cells using the modified Blyszczuk protocol (See Figure S1). 300 IPCCs or 300 pancreatic islets isolated from CBA mice were transplanted under the kidney capsule of syngeneic (CBA, H2k) or allogeneic BL/10, H2b) recipient mice and the grafts excised on day 1 or 3 days post-transplant. Half the graft was taken for immunofluorescence and half to analyse intra-graft gene expression by Q-PCR. (0.45 MB PDF) [file pone.0010965.s002.pdf]

**A**

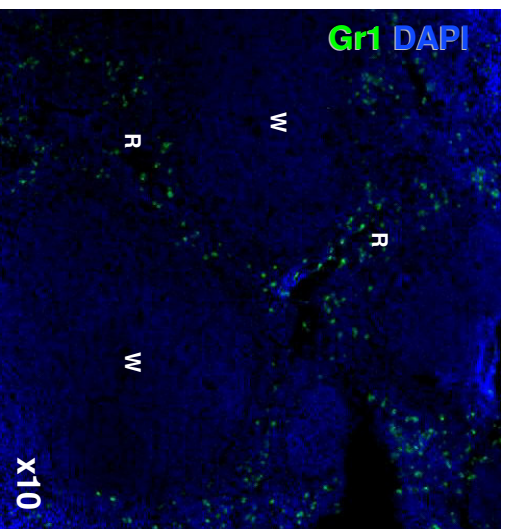

**B**

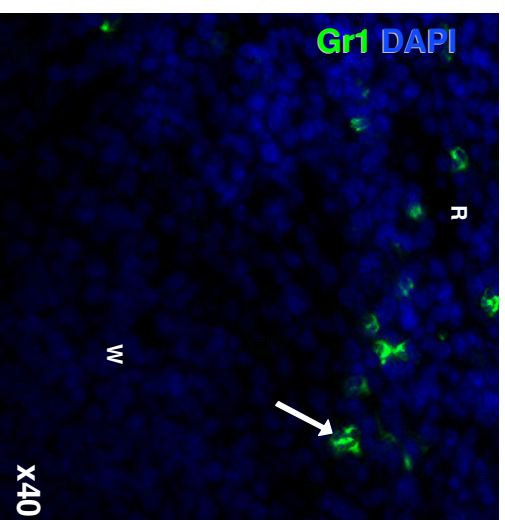

**C**

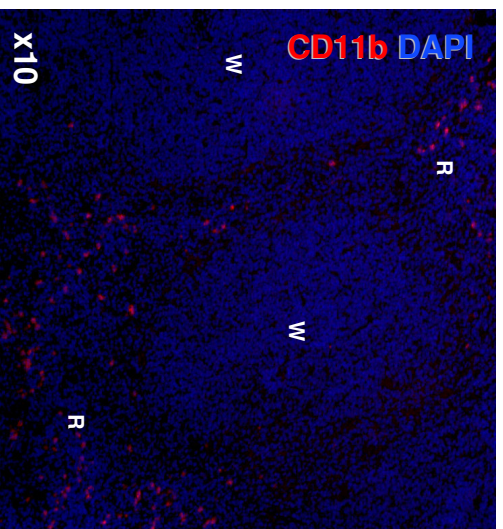

**D**

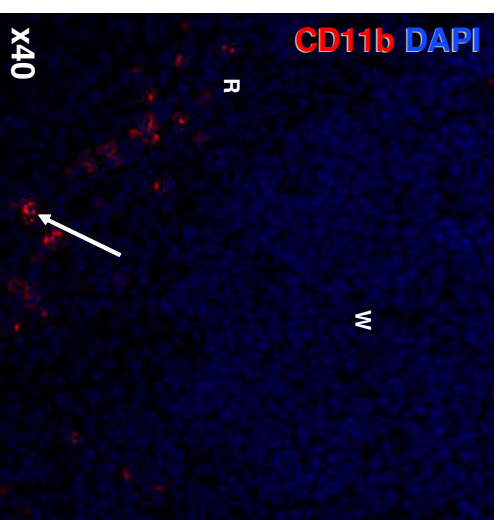

**Supplemental Figure 3**

Supplement: Figure S3 — The spleen is a source of Gr1+ and Mac1+ cells. In the study, spleen sections were used as a positive control for staining of Gr-1 and Mac-1 as the spleen is known to contain neutrophils and macrophages within the area called red pulp, labelled R in panel A. Staining of Gr-1 within the red pulp was evident at x100 (A) and x400 (B) original magnifications (original magnification calculated from use of a 10x eyepiece and a 40x objective lens). The area of the photograph labelled W in panel A corresponds to the T and B cell zone of the spleen, also called the white pulp. An arrow in B points to a close up of a Gr-1+ cell with a multi-lobed nucleus. Mac-1 staining can also be seen clearly at x10 (C) and x40 (D) original magnifications. These photographs are representative of n = 8 experiments. (0.31 MB PDF) [file pone.0010965.s003.pdf]
