# Supplementary material for: Characteristics of the Early Immune Response Following Transplantation of Mouse ES Cell Derived Insulin-Producing Cell Clusters
Source: PLoS One. 2010 Jun 4;5(6):e10965. doi: 10.1371/journal.pone.0010965 (PMC2881030; doi:10.1371/journal.pone.0010965)
Supplement: Table S1 — Primer sequences for SYBR Green Q-PCR. (0.03 MB DOC) [file pone.0010965.s004.doc]

| **Gene** | **primer sequences** |
| --- | --- |
| Gro-β/CXCL2 | for: TCCAGAGCTTGAGTGTGACGC |
|  | rev: TGGATGATTTTCTGAACCAGGG |
| CXCL10/IP-10 | for: GAAATCATCCCTGCGAGCCT |
|  | rev: ttgatggtcttagattccggattc |
| Lix/CXCL5 | for: GCTGCCCCTTCCTCAGTCAT |
|  | rev: CACCGTAGGGCACTGTGGAC |
| MCP-1/CCL2 | for: ttcacagttgccggctgg |
|  | rev: tgaatgagtagcagcaggtgagtg |
| MIP-1α/CCL3 | for: ctgtaccatgacacttgcaacca |
|  | rev: tcttccggctgtaggagaagc |
| MIP-1β/CCL4 | for: ttctcttacacctcccggcag |
|  | rev: gtactcagtgacccagggctca |
| HPRT | for: atcattatgccgaggatttggaa |
|  | rev: ttgagcacacagagggcca |
