# Supplementary material for: Characteristics of the Early Immune Response Following Transplantation of Mouse ES Cell Derived Insulin-Producing Cell Clusters
Source: PLoS One. 2010 Jun 4;5(6):e10965. doi: 10.1371/journal.pone.0010965 (PMC2881030; doi:10.1371/journal.pone.0010965)
Supplement: Table S2 — Primer sequences for TaqMan Q-PCR. (0.03 MB DOC) [file pone.0010965.s005.doc]

| **Gene** | **primer AND PROBE sequences** |
| --- | --- |
| IL-6 | for: TCCAGAAACCGCTATGAAGTTCC |
|  | rev: GTCACCAGCATCAGTCCCAAG |
|  | probe: CTCTGCAAGAGACTTCCATCCAGTTGCCT |
| TNF-α | for: TCGAGTGACAAGCCCGTAGC |
|  | rev: CTCAGCCACTCCAGCTGCTC |
|  | probe: CGTCGTAGCAAACCACCAAGCGGA |
| HPRT | for: ATCATTATGCCGAGGATTTGGAA |
|  | rev: TTGAGCACACAGAGGGCCA |
|  | probe: TGGACAGGACTGAAAGACTTGCTCGAGATG |
